# The Pre-Cancerous Cervix*Read before the Southwest Texas District Medical Society, March, 1917.

**Published:** 1917-04

**Authors:** S. A. Cunningham

**Affiliations:** San Antonio, Texas


### The Pre-Cancerous Cervix.*

BY S. A. CUNNINGHAM, M. D., SAN ANTONIO, TEXAS.

If recognized very early and properly treated would reduce the mortality of cervical cancer from about 95 per cent to practically none.

As we all have to acknowledge the fact that the time to conserve the life of these unfortunate women is before they have developed the cancerous stage.

I think early symptoms and diagnosis is by far the most important feature to be considered in handling the above condition.

From the statistics of many competent observers on this subject we learn that cancer of the uterus is the most frequent site of the disease; and that cancer of the cervix is the most frequent location in the uterus. It occurs usually between the ages of 35 and 45, but may appear anywhere from the ages of 30 to 70. As a general thing these women are the mothers of four or five children, having enjoyed previous good health.

Cervical lacerations with subsequent ectropion and erosion is frequently mentioned as predisposing factors and there is no doubt in my mind that the preliminary irritation is one of the direct causes; in the adenomatous variety chronic inflammation of the endocervical glands must play an important role.

From a pathological standpoint two separate and distinct varieties of cancer are described. The squamous cell variety, which arises from the endothelial cells covering the vaginal portion of the cervix extending a short distance up its canal. This is about seven times as frequent as the adenocarcinoma. The squamous cell variety is the one which presents itself to us in the form of the cauliflower inlfitrating mass.

The second, or adenocarcinoma, has its origin from the endocervical glands either from the endothelial cells lining the duct or from the acinous portion of the gland. Endotheliomata rarer than the other two does occur.

Cancer remains a local condition in the cervix longer than elsewhere because of the small size of the lymphatics, metastases occurring through these channels involves the pelvic glands three to five in number which are situated at the bifurcation of the iliac artery.

The metastatic tumor is of the same histological structure as the parent or primary growth. The fact that metastases is compara-

*Read before the Southwest Texas District Medical Society, March, 1917.

tively slow in developing is one of the essential reasons why early recognition is of paramount importance in the so-called precancerous cervix.

The early subjective symptoms of this malignant condition are of such a nature that they arouse no suspicion either in the mind of the patient or doctor as to the true nature of the condition, and are frequently passed over as unimportant details.

Hemorrhage at first slight in amount occurs independent of the menstrual period, it causes no inconvenience or alarm to the patient and is described by her as a mere stain.

Hydrorrhea, while not excessive, is a more or less constant feature. In the beginning the only difference between hydrorrhea and leucorrhea is in quantity and not in quality, but later on the hydrorrhea is more watery but very difficult to distinguish clinically, consequently we should be very careful to ascertain whether or not the discharge the patient is suffering from contains any blood.

The lymphatic glands of the cervix may or may not be found. As you know, in health these glands are very small, so that not finding them does not prove whether they are diseased or not, but should enlarged glands be found it is most significant with the foregoing symptoms and conditions.

Pain is conspicuous by its absence. Objectively the cervix is paler in color and firmer in consistency on account of cellular infiltration. Hydrorrhea is present which may be blood tinged. Lacerations or erosions will be self-evident. When confronted with the above symptoms we are justified and it is imperative that we remove a section from the cervix for microscopic examination.

The histological examination will determine whether local reparative measures shall be taken or the radical operation done.

A safe procedure to adopt in treating suspicious cases of the cervix would be to remove the diseased tissue by some plastic operation, the choice of same depending upon the one which has proven of greater benefit to the operator.

Personally, I prefer doing an amputation, as it gives me a better view of my field of operation, and I think I can be more certain that I have removed all of the diseased cells. Then in all cases of this type I have a histological examination made of the section that is removed. Some of our surgeons claim to have succeeded in permanently curing a large number of early carcinoma of the cervix by a high amputation of the cervix by means of the hot platinum loop.

Bryne, of Brooklyn, claimed to have had a death rate of only 19 per cent after a period of five years in 367 cases treated by him.

He also advanced the theory that the reason better results are not obtained by the cautery today is the lack of thoroughness and keeping it up by further applications when there is the least evidence of recurrence of diseased tissue. He regards the black and charred surface the best safeguard against the recurrence of the disease.

Gentlemen, I have written this article on the above subject not with the idea of presenting any new facts, but to call your attention to the vital necessity of making a more complete and thorough examination of each and every woman in the cancerous age who consults you, no matter for what condition; and make it a point to ask her specific questions as to her menstrual function or menopause. It is in this way that we will be able to make vaginal examinations and an early diagnosis, thereby reducing the vast difference of percentage of operable cases that present themselves to the clinics of this country from those of Europe, as it is stated 60 per cent of all cases of cancer of the cervix in Europe are operable, while the percentage in this country is only 38 per cent, and I feel we should adopt some means whereby this difference should be reduced, and at the same time decrease the mortality of this dreaded disease.

